# Food Choice and Dietary Perspectives of Young, Urban, Black Pregnant Women: A Focus Group Study

**DOI:** 10.3390/nu16060781

**Published:** 2024-03-09

**Authors:** Tristesse Catessa Jasmin Burton, Natasha Crooks, Lacey Pezley, Nefertiti OjiNjideka Hemphill, Yanqiao Li, Arissara Sawatpanich, Vanessa Farrow, Katherine Erbe, Nicollette Kessee, Luecendia Reed, Lisa Tussing-Humphreys, Mary Dawn Koenig

**Affiliations:** 1 Department of Pharmacy Practice, University of Illinois Chicago, 833. S. Wood Street, Chicago, IL 60612, USA; 2 Department of Human Development Nursing Science, University of Illinois Chicago, 845 S. Damen Ave., Chicago, IL 60612, USA; ncrooks@uic.edu (N.C.); yli344@uic.edu (Y.L.); asawat2@uic.edu (A.S.); vanessajfarrow@gmail.com (V.F.); marydh@uic.edu (M.D.K.); 3 Department of Kinesiology and Nutrition, University of Illinois Chicago, 1919 W. Taylor St., Chicago, IL 60612, USA; lwissl2@uic.edu (L.P.); nefertiti@nutrientinnovation.com (N.O.H.); nkesse2@uic.edu (N.K.); ltussing@uic.edu (L.T.-H.); 4 Ramathibodi School of Nursing, Faculty of Medicine Ramathibodi Hospital, Mahidol University, 270 Rama VI Rd., Ratchathewi, Bangkok 10400, Thailand; 5 Yvonne L. Munn Center for Nursing Research, Massachusetts General Hospital, 55 Fruit Street, Boston, MA 02114, USA; kerbe@mgh.harvard.edu; 6 New Moms, 5317 W. Chicago Ave., Chicago, IL 606051, USA; luecendia.reed@yahoo.com

**Keywords:** Black women, pregnancy, food apartheid, social determinants of health, food desert, food choice, Chicago

## Abstract

Black pregnant women in Chicago are disproportionately affected by maternal morbidity and mortality and are more likely to reside in neighborhoods that experience greater economic hardships and food apartheid than any other race/ethnicity. Addressing social determinants of health such as structural inequities, economic environment, and food apartheid issues may provide insights into eliminating Black maternal morbidity and mortality disparities. This study explores food choice determinants and dietary perspectives of young, urban, Black pregnant women. Two audio-recorded focus groups were conducted in Chicago, IL between March 2019 and June 2019 to discuss pregnancy experiences and factors affecting maternal nutrition. Thematic analysis was used to identify the codes, themes, and subthemes of the data. Data analysis was guided by the Social Ecological Model (SEM) as a theoretical framework. Eleven, young, Black women were recruited. Three major themes were discussed across the SEM levels that influenced food choice including food access, stress and family influences on eating, and the need for nutritional education during pregnancy. These choices were primarily rooted in the detrimental effects of food apartheid experienced within the participants’ neighborhoods. Therefore, acknowledging, understanding, and addressing food apartheid and its impact on Black maternal health disparities is needed in clinical practice, research, and policy change.

## 1. Introduction

Consistent with United States (U.S.) trends, Black women in Chicago experience higher maternal morbidity and mortality at rates nearly three- and six-times higher, respectively, than White women [1]. Additionally, Black women in Chicago are more likely to reside in communities with greater economic hardships, more likely to live under food apartheid, and experience the majority of the maternal morbidity and mortality burden [1,2]. Several U.S. initiatives such as the Black Maternal Health Week and Black Maternal Momnibus Act (Rep. Underwood, 2021) along with research funds exceeding USD 128 million in 2022–2023 emphasize the significant role of the social determinants of health (SDOH) on these disparities [3,4,5]. Addressing SDOH factors includes examining the intersection between racism, economic environment, structural inequities, poor health care, and issues relating to food apartheid (i.e., food deserts) including insufficient access to healthy and affordable food [1,6].

Inadequate maternal nutrition during pregnancy has short- and long-term implications for both mother and baby. For the baby, poor maternal nutrition is associated with an increased risk of large development for gestational age [7,8,9], neural tube defects [8,10], preterm birth [8,11], and future development of type 2 diabetes and obesity [12,13]. For the mother, an unbalanced diet with limited fruit and vegetable consumption and high consumption of fried food is associated with an increased risk of developing gestational diabetes [14], hypertension [15,16], postpartum weight gain [16], complications during pregnancy [9,17], and future development of cardiovascular disease [18].

Food choice is multidimensional, complex, and influenced by interrelated intrinsic and extrinsic determinants [19,20,21]. One’s built environment, social and cultural dynamics, and context (e.g., social situations, time allotment, and physical settings) all impact food choice [22]. Furthermore, food choices are influenced by marketing and economic factors, along with one’s habits, goals, knowledge, and perceptions around healthy eating [19].

Little is known about what influences food choice and dietary intake of young, urban, pregnant Black women. To our knowledge, only two previous qualitative studies examined food choices, barriers, and facilitators for healthy eating in this population [8,23]. These studies highlight that urban Black pregnant women desire to eat healthy, but food cravings, appetite, and taste influence their food choices. Additionally, this population reports that their neighborhoods and built environment are structured with a surplus of fast-food restaurants and food high in fat, while barriers such as time, cravings for unhealthy foods, and finances impinge on making healthy dietary decisions [8,23]. Furthermore, the Institute of Medicine has called for more research focusing on minoritized women that provides improved and culturally tailored care for healthy nutritional intake and weight gain during pregnancy [24,25]. More qualitative research is needed to better understand the determinants of food choice, motivators, and barriers to healthy eating for Black pregnant women. The objective of this study is to explore determinants of food choices and dietary perspectives of healthy eating from young, urban Black pregnant women.

## 2. Materials and Methods

### 2.1. Theoretical Framework

The Social Ecological Model (SEM) was used as a theoretical framework to guide the data analysis [26,27]. The SEM is a comprehensive, multi-level framework encompassing five key elements. *Intrapersonal* influences are individual factors that influence behaviors such as beliefs, personality, attitude, and knowledge. *Interpersonal* influences include the relationships with family, friends, and peers that develop social identity and make up social support systems. *Organizational* influences are rules, policies, and regulations that may promote or constrain behavior in informal structural settings such as schools or workplaces. *Community* influences are behaviors in social norms, standards, or networks that occur formally or informally in community-like settings. *Societal* influences are broader factors such as social policies, economics, and norms that encourage or deter specific behaviors [27]. We used the SEM to understand the determinants of food choice and the barriers and motivators to eating healthy across multiple SEM levels. Previous studies used the SEM as a framework to improve and understand dietary intake among Black, low-income [28], and diverse pregnant women [29].

### 2.2. Participants

Participants were recruited from New Moms, a non-profit organization on the West Side of Chicago that provides prenatal and family support programming, job training, and transitional housing to improve the well-being of young, low-income mothers. New Moms is located in the Austin community, one of the largest geographical and populated neighborhoods of Chicago with 75% of adults identifying as Black with the highest adverse maternal–infant health outcomes [1]. A convenient sample of pregnant women attending prenatal classes at New Moms were approached between March 2019 and June 2019 to participate in focus group discussions related to stress and pregnancy (IRB # 2016-0662).

### 2.3. Data Collection

Two audio-recorded focus group discussions, lasting 60–90 min, were held at New Moms with five and six participants in each session, respectively. Dr. Nefertiti OjiNjideka Hemphill (NOH), who identifies as a Black cis-gendered woman with experience in focus group data collection, led the discussion and had no prior relationship with the participants. Questions were asked about participants’ pregnancy experiences with nutrition, with clarifying questions asked as needed. Audio recordings were transcribed verbatim.

### 2.4. Data Analysis

Data analysis was driven by Braun and Clarke’s [30] thematic analysis framework to better understand the perspectives related to nutrition, food choice, and food environment of the study participants. This thematic analysis was conducted by MDK, NC, LBP, AS, YL, VF, and TB as a collaborative research team consisting of students, postdoctoral fellows, and faculty experts in health disparities and maternal health research. NC, VF, and TB identify as Black cis-gender women, MDK and LBP identify as White cis-gender women, and YL and AS identify as Asian cis-gender women. We addressed potential biases by recognizing how our own experiences and beliefs shaped our perspectives on racism, ageism, sexism, and classism, and how the intersection of these identities may influence our interpretation of analyzing the participants’ experiences [31].

To begin this analysis, each team member reread the focus group transcripts. The data were individually coded to generate initial codes. Next, the team met weekly to discuss discrepancies and reached a consensus on the final codes to be grouped and created themes for each quote. The research team meetings allowed for discussions on the analysis, authenticity of coding, and thematic development to ensure the validity of the analysis [30]. Three major themes were developed from the data, with defining subthemes. Exemplary quotes for each theme were provided with Focus Group number [*FG #*]; because the surveys were completed anonymously, we could not report the corresponding ages for the quotes. 

The data of this study is reported using the consolidated criteria for reporting qualitative research (COREQ) checklist [32] (Table S1). 

### 2.5. Research Trustworthiness

Research Trustworthiness (i.e., creditability, transferability, and confirmability) was ensured using the criteria detailed by Lincoln and Guba [33]. Credibility was established by continued engagement with the data, including re-reading the transcripts and frequent discussions on the meaning, context, and quote selection. Data analysis took three months and consisted of regular team meetings, coding, and revising codes. The transferability of findings was supported by the detailed description of the methodology and contextualization of narratives from the participants. Confirmability was accomplished through discussions on potential biases, along with peer debriefing and working as a research team to ensure accurate data representation.

## 3. Results

A total of 11 participants consented to participate, ranging from 18 to 25 years old (Table 1). Participants described three major themes highlighting specific approaches and obstacles related to food access, stress and family influences on eating, and the need for nutritional education during pregnancy (Table 2).

### 3.1. Food Access

Participants shared their perspectives on local food access, which limited their ability to eat well-balanced meals. Subthemes of food access included food apartheid, preference for convenient food options, food expenses, diet restrictions, and repetitive options in food selection during pregnancy involving influences at the community/societal and intrapersonal SEM levels.

#### 3.1.1. Food Apartheid

Participants emphasized their difficulty in accessing affordable and healthy food options in their neighborhoods, especially at unconventional times. As one participant shared her experience: “*If I wanna get up and go get food at 2 o’clock in the morning, ain’t no healthy restaurants gonna be open.*” *(FG 2)* Participants voiced their preference to shop for groceries in different neighborhoods with better food options. As one participant shared, “*I don’t even shop in this neighborhood. I shop [in] west loop…there’s a Pete’s [grocery store].*” *(FG 2)* Participants indicated that their immediate neighborhood lacked sufficient grocery stores or establishments that offer healthy food choices, motivating the participants to travel beyond their immediate neighborhood to an area with more suitable options and healthier alternatives. In response to limited food selections, participants reported creating their own healthy alternatives. One participant highlighted her preference for making nutrient-rich homemade beverages as a means of avoiding unhealthy foods:

*“Because I live by a donut shop, [they] have the mango smoothies...like that’s literally yogurt…I wake up in the middle of the night and I’m hungry, I’m making a smoothie. As for a chicken sandwich or four-piece nuggets, I don’t eat that.”*

*(FG 2)*

Participants shared their challenges in accessing nutritious and affordable food in their neighborhoods. Their collective experiences reinforce the impact of geographic location on disparities in nutrition.

#### 3.1.2. Preference for Convenient Food Options

Convenience emerged as an essential factor in healthy eating. Participants indicated that when pressed for time to prepare their meals, they were more likely to settle for a quicker and more readily available option. For example, one participant mentioned how the time required to prepare, cook, and serve a healthier meal influenced their food choice: “*It’s easier to cook a regular hamburger than it will for me [to cook] a baked one.*” *(FG 2)* Black women expressed frustration with the discrepancy between their intentions and actual eating habits, acknowledging the potential negative impact on their health. One participant stated,

*“They [doctors] be like okay eat something more healthy, but at the time I was working. So, I’m sitting here eating what y’all telling me not to eat. That’s messing up my health.”*

*(FG 2)*

These experiences highlighted the struggles young pregnant Black women face in prioritizing healthy eating in the face of time constraints and the lure of fast-food convenience:

*“When I’m out and I’m ripping and running and I don’t like pack me a lunch or something, it’s like it’s easier to just stop at McDonald’s or somewhere and get something quick.”*

*(FG 1)*

Other participants revealed their internal conflicts between their intention to make healthier eating choices by engaging in meal prepping and selecting healthier options such as fruits or yogurt. However, participants reported that when hunger struck and time constraints were present, they found it easier to succumb to the temptation of fast food. This dichotomy between the aspiration to maintain a nutritious diet and the tendency to indulge in unhealthy fast food options during moments of hunger is expressed by one participant:

*“I made about 75% of prepping my stuff than getting like regular foods say if like, say I had an early doctor’s appointment… I was maybe like you cooking something or you know I’ll probably grab an apple or my yogurt…but then I’m hungry so then I’m like okay let’s go to McDonald’s. Like I don’t even like to eat McDonald’s other than chicken sandwich other than burgers…so I’ll stop and just get that.”*

*(FG 1)*

Participants highlighted the challenges individuals faced in balancing their convenience, time restrictions, and health-conscious eating choices. The convenience and accessibility of fast food often outweighed the desire to eat healthier, particularly when individuals were busy or unable to obtain healthier options.

#### 3.1.3. Food Expenses

Participants expressed their concerns about the higher cost of nutritious and healthier foods compared to less healthy alternatives, “*Healthier food is always more expensive.*” *(FG 2)* Participants reported a financial burden of prioritizing healthy eating. Additionally, participants voiced concerns about the higher costs of quality protein foods compared to foods that are filling but unhealthy and cheap from fast food restaurants. The financial strain felt by these individuals is at odds with their desire to make healthier food choices.

#### 3.1.4. Diet Restrictions

Participants with dietary restrictions reported limited food choices that transcended intrapersonal and community/societal SEM levels. Vegetarian participants expressed frustrations about the limitations in the variety of food available to them in contrast to those who consume meat: “*It’s price and, because with me like y’all have chicken, pork, beef, and I got like very narrow range of things that can eat.*” *(FG 2)* One participant expressed frustration about the repetitive nature of available choices,

*“Like this with shrimp in it, this with you know what I mean it’s repeating with the same thing. You can do a pork chop, you can do a pork loin, pig, you can do a steak. You can get so many options.”*

*(FG 2)*

Some vegetarian participants underscored the challenges of relying on restaurants and emphasized the importance of preparing meals at home. The lack of diverse food options and limited dining venues presented significant barriers for vegetarians, leaving them to resort to snacking. This struggle was summed up by the frustration expressed by one participant.

*“You can’t like really go to a restaurant and get food. It’s more like you have to cook and you have to, and if you don’t well I better got snacks cuz it looks like I ain’t gonna eat.”*

*(FG 2)*

Limited food choices and access posed a challenge to participants with dietary restrictions, specifically vegetarians. Participant experiences demonstrate the importance of eliminating disparities in food access and selection to support individual dietary preferences.

#### 3.1.5. Repetitive Options

Since participants were dissatisfied with the lack of variety in their food choices, they reported consuming the same foods or eating at the same restaurants repeatedly. One participant shared that they were aware of the repetitive nature of their diet: “*When I started…I noticed that I was eating restaurant food for a week straight and I was like wait a minute.*” *(FG 1)* These thoughts and experiences shared by the participants exemplified the participants’ boredom and fatigue with the absence of variety in their meals. Participants reported experiencing pleasure from seeing food that looked colorful and were interested in incorporating these colorful foods into their diet. One participant expressed a desire for more education on how to vary the preparation of foods:

*“It would be, first of all colorful. Colors make everything better. It would have different options and different things you can do with oh a spinach. How many different options can you do instead of just a spinach salad or a spinach dip?”*

*(FG 2)*

Participants indicated that they were open to variety in their food choices and enjoyed when they were exposed to different types of foods.

*“I need some healthy foods that taste good, and I need some options. Like I need to know like what’s out there. Because like [Participant], I think she’s vegetarian…she be bringing new stuff so we tried and I’m like okay well let me go to the store and see if I can get this.*”

*(FG 2)*

Participants enjoyed experiences where they were exposed to different foods and would make plans to buy these items at the grocery store.

### 3.2. Stress and Family Influence on Eating

Another theme identified by participants was the emotional relationship they had with their food choices and eating patterns, commonly influenced by stress and family pressure experienced at the intra/and interpersonal SEM levels. Subthemes included pressure to lose weight and appetite changes due to stress. Many participants described trying to overcome stress and family pressures to make conscious decisions about their food choices and eating habits.

#### 3.2.1. Pressure to Lose Weight

Participants described guilt or shame for not eating as healthily as they should, which created additional stress. One participant described the importance of food in losing weight. She stated, “*weight loss is 80% what you eat and 20% what you do. You can go to the gym every single day, but if you go home to fried chicken then.*” *(FG 2)* Other participants reported how family members’ relationship with food impacted their family relationships. One participant stated, “*My mom was on this weight loss thing, and so she’s eating healthy so like every time she come over, she gain a little bit of her weight back. So, I get blamed for it because of my eating habits.*” *(FG 2)* Participants also felt that the advice from their medical team on gestational weight gain and healthy eating disregarded contextual barriers and was not sensitive to the participants’ needs. One participant described pressure to lose weight from her doctor in regard to health conditions:

*“She’s [Doctor] like, ‘Oh it’s your weight. And you need to lose weight’ I’m like, this happened before I got pregnant. Like, I gained like, I gained 80 pounds pregnant with her. So, I got my smallest I ever was before I got pregnant, but this was before I got pregnant, this was before I was big.”*

*(FG2)*

Participants reported that family and medical providers suggested losing weight during pregnancy which created stress for the study participants. Instead of suggesting losing weight, participants preferred nutritional counseling and sensitivity around contextual barriers that influence food choices, such as family dynamics and limited food access.

#### 3.2.2. Appetite Changes Due to Stress

Participants reported they often over or under-eat when they experience stress. Participants stated because of stress “*I don’t eat,*” “*I lose my appetite,*” *or* “*I eat a lot.*” *(FG 1)*. One participant described the impact of her mental health on her diet, “*If I’m mentally stressed, I don’t have enough energy to eat. And then, depending on like what it is, I don’t eat well.*” *(FG 2)* Another participant attributed losing weight attributed to her stress:

*“When I was pregnant, I lost 65 pounds throughout my pregnancy…that’s cuz I was stressed and depressed like people don’t understand. Like I couldn’t function when I was pregnant. Like my man was not there at all. The only thing that saved me throughout my pregnancy was my mama and... that was it cuz I promise you.... that’s how bad it was.” *

*(FG 2)*

Notably, the presence or absence of social support at times affected the relationship between stress and eating. Participants reported engaging in emotional eating (whether over or under-eating) to deal with feelings instead of to satisfy their hunger. Participants reported that support structures were critical to their success in overcoming this.

### 3.3. Need for Nutritional Education during Pregnancy

It was clear that participants were aware of the benefits of healthy eating during pregnancy, and yet, integrating healthy eating into their daily lives was a challenge. Two subthemes emerged that highlighted the need for nutrition counseling during pregnancy at the organizational SEM level. These subthemes included awareness of healthy eating and perspectives on healthy food and developing healthy eating patterns during pregnancy.

#### 3.3.1. Awareness of Healthy Eating and Perspectives on Healthy Food

Many participants described feeling empty after eating or did not know how much food to consume during pregnancy to achieve satiety. One participant reported feeling hungry after eating healthy food and needing more to satisfy themselves, “*healthy foods are not as filling as regular foods.*” *(FG 2)* Participants recognized physical changes occurring in their bodies and how their hunger increased because of their pregnancy.

*“I get upset because I just look at it like this is not gonna fill me up and then you’ll eat it and you’ll still be hungry I swear. Some would fill me up but most times I just be like this is not enough for me.”*

*(FG 2)*

Participants requested information on food options that would provide satiety. They reported struggling with feeling hungry and strategized by eating smaller more frequent meals. Despite this, many participants reported feeling continued hunger.

*“I would wanna learn like things to fulfill me...I could eat 10 bowls of greens straight nonstop...but it’s like if I get up and eat an apple, I’ll literally be hungry 10 min later. I’m constantly eating and I’m like okay I eat an apple, I eat some carrots with peanut butter and I get like um like a yogurt or whatever but it’s like that stuff don’t fill me up.”*

*(FG1)*

Participants also expressed concerns about taking in adequate amounts of essential nutrients, vitamins, and minerals during pregnancy and balancing this with a healthy weight gain. One woman reported eating one meal a day and eating smaller snacks, like fruit, throughout the day to achieve their calorie intake. Another participant shared, “*I worry about eating enough. Yeah, like I only eat one full meal a day. But besides that, one meal I’ll eat a lot of fruit.*” *(FG1)* Participants’ confusion regarding the food needed during pregnancy led to their desire for more dietary education from a trusted source.

Participants stated the need for more nutrition counseling and education during pregnancy, “*I would say the basic of course like the healthy eating or like dark green leafy stuff or like protein...stuff that would help you out especially with like constipation*.” (FG 1) Satiety, amount of food required during pregnancy, and frequency of eating were highlighted by the participants. Although participants recognized and adopted healthy eating habits, they experienced feeling unsatisfied and lack of satiety after consuming healthy meals.

#### 3.3.2. Developing Healthy Eating Habits

Pregnancy provided strong motivation for the participants to develop healthy eating habits. Most participants stated that when they got pregnant, they made changes to eat healthy:

*“In the beginning [of pregnancy]…I was eating healthy cuz I’m a dancer, but then of course you have your days where you want some Popeyes. But ever since I got pregnant it literally knocked all that out, and I was eating healthy.”*

*(FG 1)*

To maintain healthy eating habits, one participant explained that she prioritized natural flavors and reduced her intake of salt, sugar, and processed foods:

*“I actually got healthier… I’m able to eat rice without butter or sugar or salt and pepper, nothing, just plain. I’m able to eat my vegetables plain without any extra stuff with it. I eat salad with no salad dressing.”*

*(FG 1)*

Participants understood that their food choices were influenced by their environment which dictated their food access at the communal or societal level. Additionally, stress and family influence on eating affected participants’ food choices at the inter/intrapersonal levels. Although participants understood the benefit of healthy eating during pregnancy, they highlighted the need for nutritional education during pregnancy which can be implemented at the communal and societal levels.

## 4. Discussion

This qualitative study highlights the nutritional perspectives of young, Black, pregnant women residing on the West Side of Chicago. Findings emphasized three themes related to participants’ determinants of food choice and dietary perspectives during their pregnancy: food access, stress and family influence on eating, and the need for nutritional education during pregnancy. Using the SEM as a theoretical framework, we identified how these three themes transcended each level within this model. Participants’ food choice was influenced at the intra/interpersonal level by stressful experiences and relationships with family members and at the community/organizational/societal levels due to food apartheid-related issues. These influences highlight the impact of their food environment on food choices within the neighborhoods where the participants reside.

To our knowledge, only a few studies discuss food choice and linkages to food apartheid, food deserts, or food inequities and their impact on pregnant Black women [8,23,34,35,36]. These studies reported that built food environments are significantly associated with pregnancy morbidity [34,36] and impede food choice [35]. Specifically for Black women, Reyes et al. (2013) examined motivators and barriers to healthy eating in 21 overweight Black pregnant women in Philadelphia [8]. Their study participants discussed how they wanted to eat healthy; however, taste, cost, and convenience drove their food choices toward foods that were high in fats and sugar [8]. Another study by Groth et al. (2016) in low-income pregnant Black women also noted that food cravings, taste, and appetite influence food choice; however, it did not highlight access as a limiting factor [23]. Similarly, our study participants discussed the influence of taste and satiety on their food choices.

Our study participants explained the significant impact of their built food environment with convenience and access to healthy food options as nutritional determinants that negatively impacted their food choices. Chicago is historically known for its “redlining” and segregated communities that promoted the disenfranchisement of Black individuals’ food environment. As a result, residents resort to what is easy, accessible, and appeases their taste, such as unbalanced calorically dense options at fast food locations or convenience stores. In addition to the community/organizational/societal level effects on the nutritional environment, it was apparent that the intra- and interpersonal level affected food choices for our study participants. Our study participants discussed their relationship with their mothers and the impact this relationship had on their food choices. For example, some participants’ mothers emphasized healthy eating during pregnancy indirectly (by commenting on weight gain) or directly (cooking healthier food options); however, other participants preferred not to eat meals prepared by their mothers because it was not filling or addressing their craving. This relationship between cravings, food preferences, and influence from the participants’ mothers prompted more stress and shame related to gestational weight gain and diet for some of our study participants.

Several studies highlight that Black women are considered gatekeepers of food practices [37,38], learning recipes from their mothers and grandmothers. However, our study participants did not mention the role of intergenerational food knowledge, potentially due to the young age of the participants and undisclosed family dynamics. However, stress heavily disrupted the eating habits and food choices of our study participants, but this was not observed in previous studies [8,23].

Our findings support the work of others that show Black pregnant women want to eat healthier [23] and are aware of healthy eating effects on their babies in utero development [8], although they are faced with limited awareness, behavior change techniques, and support on implementing portion control and preparation of healthy, satisfying meals. Furthermore, pregnancy is a life event that may change the direction of food choice and dietary intake behavior [39]. As in previous studies [40,41,42], our participants discussed that pregnancy promoted healthy eating and better food choices; however, they struggled to find resources to support this change. Furthermore, during pregnancy, women are typically motivated to adopt healthier lifestyles and are under more frequent medical care [43]. Therefore, pregnancy is a potential “window of opportunity” for implementing techniques that support making healthier food choices and improving nutritional intake that may be sustained throughout the lifespan [43]. However, these techniques need to be personalized to the individual and cultural needs of pregnant Black women.

Our participants desired nutrition education through a comprehensive approach embedded in doctor’s visits where their medical team works with Black mothers to understand contextual barriers at each SEM level that inhibited their success in their diet and food choices. Nutritional counseling, especially guidance in preparing nutritious and flavorful meals, may help meet our study participants’ dietary needs while satisfying their hunger. This sentiment resonates in other studies with diverse low-income women, including Indigenous women [35,44,45,46] and Black pregnant women and mothers [47], highlighting the need for tailored nutritional education that supports healthy eating to address these barriers.

To our knowledge, this qualitative study is the first to explore determinants of food choice of urban, young, Black pregnant women. However, this study is not without limitations. Our study included a small sample size of Black women, which cannot be generalized to other populations. We focused on a subset of young, single, Black women with low socioeconomic status, on the West Side of Chicago, limiting our generalizability and ability to reach data saturation. Yet, these data demonstrated enough redundancy to identify repeated themes. Additionally, this study was limited in that we did not ask about specific nutrition public assistance programs such as the Supplemental Nutrition Assistance Program and Women’s Infant and Child program, which may provide resources to motivate or impede healthy food choices. In the qualitative nature of this study, we did not collect details on the participants’ demographic background, including BMI, gestational weight gain, division of household tasks, and parity, which may play a significant role in decision-making regarding eating behavior and food choice. Nonetheless, our results are meant to provide greater insight into Black maternal health and determinants that impact food choice which can be used to develop interventions for this population targeting the barriers identified in this study.

### Implications for Practice, Research, and Policy

This research further explains how certain determinants influence food choices for young, urban, Black pregnant women, highlighting areas to implement in clinical practice, research, and policy. Nutrition education and counseling must be individualized in content and approach based on the patient’s experience. Additionally, healthcare providers should listen to Black women’s stories and experiences to understand their nutritional needs and support. More research on determinants of food choice for Black pregnant women needs to be community-driven with input from Black women and inclusive of the diversity displayed within this population (i.e., varying socioeconomic status). Additionally, there is a critical need for more Black women scientists in the academia to support research that mitigates Black maternal health disparities [48]. Inclusive and collaborative research can enforce targeting maternal Black health disparities from multiple angles.

Self-determination and resiliency have led to food justice initiatives targeting food inequities in Black communities in Chicago through food sovereignty efforts and urban farming. Food sovereignty is one strategy to dismantle food apartheid by committing to food justice advocacy and situating people and communities rather than corporations in the control of the entire food system, from production to consumption of food [49]. Chicago organizations have implemented food sovereignty initiatives such as Food as Medicine approaches, farmers markets held in Black neighborhoods, and education on healthy eating, cooking classes, and local healthy food availability [50,51,52]. Yet, more financial resources for food justice initiatives, policy changes to support autonomy in healthy food choice, and inclusive and targeted nutritional health care are still needed to address systemic food inequity issues in Chicago that affect Black pregnant women.

## 5. Conclusions

Young, urban, Black pregnant women are affected by the determinants of food choice at multiple levels that influence their food choices. This study illustrates that the multiple factors at the inter/intrapersonal levels, as well as community, organizational, and societal levels rooted in food apartheid, contribute to poor food choices and low-quality food environments. Acknowledging and naming food apartheid and intra/interpersonal influences on Black women’s food environment aligns with the evidence that understanding structural determinants and their impact are essential in making radical changes toward the root causes of Black maternal health disparities.

## Figures and Tables

**Table 1:** Demographic characteristics of focus group participants.

Participant	Age (Years)	Race	Ethnicity	Marital Status	Income	Employed	Education
**Miss A**	23	Black	Not Hispanic	Single	USD 520/month	Not employed	High school
**Miss B**	23	Black	Not Hispanic	Single	none	Not employed	High school
**Miss C**	22	Black	Not Hispanic	Single	USD 16/hour	Full time	Some college
**Miss D**	25	Black	Not Hispanic	Single	USD 15/hour	Full time	High school
**Miss E**	22	Black	Not Hispanic	Single	USD 620/month	Full time	High school
**Miss F**	22	Black	Not Hispanic	Single	USD 2250/month	Full time	Some college
**Miss G**	20	Black	Not Hispanic	Single	none	Not employed	High school
**Miss H**	21	Black	Not Hispanic	Single	USD 12.50/hour	Full time	High school
**Miss I**	19	Black	Not Hispanic	Single	USD 14/hour	Full time	High school

Note: No data are available for Miss J and Miss K, respectively, because they attended the prenatal class only.

**Table 2:** Identified themes and subthemes from focus group discussions.

Theme	Subtheme	Definition
**Food Access**	*Food Apartheid*	Difficulty in accessing healthy food options based on where they live and structural and systemic issues affecting food access. Factors such as the location of grocery stores, farmers’ markets, and restaurants can impact the availability and affordability of healthy food options for pregnant women.
	*Preference for convenient food options*	The challenge of balancing busy schedules with healthy eating habits. It recognizes that sometimes, due to time constraints or lack of meal planning, pregnant women may resort to convenient food options such as fast food or processed snacks.
	*Food Expenses*	Increased cost of food as a barrier to healthy eating.
	*Dietary restrictions*	The struggles faced by Black pregnant women who follow a vegetarian diet or who have health issues due to limited options available to them.
	*Repetitive Options*	Challenges in the limited variety of food options, awareness of diverse recipes, and restaurant availability that limit the selection of healthy food choices.
**Stress and Family Influence on Eating**	*Pressure to Lose Weight*	The tendency of individuals to attribute their weight gain to their eating habits or dietary choices, leading to feelings of guilt and shame.
	*Appetite Changes Due to Stress*	Appetite changes or desire to eat decreases or increases due to stress or as a mechanism to cope with difficult situations.
**Need for Nutritional Education During Pregnancy**	*Awareness of Health Eating and Perspectives on Health Food*	Understanding healthy eating and its impacts on baby and maternal health still has challenges in terms of healthy eating due to still feeling hungry after a healthy meal/snack, or worrying about portion sizes, which highlight the need for more nutritional education.
	*Developing Health Eating Habits*	Adaption of healthier eating practices because of pregnancy, but it is not easily maintained, which emphasizes the need for sustainable nutritional education.

## Data Availability

The data for this study are not publicly available, but summaries are either included in the manuscript or available from Mary Dawn Koenig, Department of Human Development Nursing Science, University of Illinois Chicago, 845 S. Damen Ave. MC 802, Chicago, IL 60612, USA, marydh@uic.edu. The data are not publicly available due to it containing information that could compromise the privacy of the particiipants included in this study.

## Supplementary Materials

The following supporting information can be downloaded at: https://www.mdpi.com/article/10.3390/nu16060781/s1, Table S1. COREQ (COnsolidated criteria for REporting Qualitative research) Checklist.
